# Head pleomorphic sarcoma showing murine double minute 2 amplification without a well‐differentiated liposarcoma component in a pediatric patient

**DOI:** 10.1002/cnr2.1774

**Published:** 2022-12-26

**Authors:** Mitsuko Akaihata, Ikuko Takahashi, Yuko Kakuda, Takuya Kawata, Takashi Mukaigawa, Testuro Onitsuka, Shigeyuki Murayama, Yuji Ishida

**Affiliations:** ^1^ Division of Pediatrics Shizuoka Cancer Center Shizuoka Japan; ^2^ Division of Pathology Shizuoka Cancer Center Shizuoka Japan; ^3^ Division of Head and Neck Surgery Shizuoka Cancer Center Shizuoka Japan; ^4^ Division of Head and Neck Surgery Mishima Central Hospital Shizuoka Japan; ^5^ Radiation and Proton Therapy Center Shizuoka Cancer Center Shizuoka Japan

**Keywords:** liposarcoma, MDM2 amplification, pediatric, pleomorphic sarcoma

## Abstract

**Background:**

Murine double minute 2 (MDM2) is an oncogene that inhibits p53, leading to decreased apoptosis. Sarcomas showing MDM2 amplification are rare among pediatric patients.

**Case:**

A 14‐year‐old boy presented with pleomorphic sarcoma of the head showing MDM2 amplification without a well‐differentiated liposarcoma component. Although chemotherapy was initially performed to reduce the tumor size before surgery, the tumor did not shrink. The patient underwent complete surgical resection. Microscopic examination revealed a positive surgical margin; thus, postoperative proton‐beam radiotherapy was performed. 3 years after the therapy, no sign of recurrence was observed.

**Conclusion:**

Macroscopic surgical resection combined with adjuvant postoperative radiotherapy was effective against MDM2‐amplified pleomorphic sarcoma refractory to neoadjuvant chemotherapy in a pediatric patient.

## 1 INTRODUCTION

Murine double minute 2 (MDM2), which is located at 12q15, inhibits p53; this results in decreased apoptosis.^1^ Well‐differentiated liposarcoma (WDLPS) and dedifferentiated liposarcoma (DDLPS) are associated with amplification of the chromosomal 12q13‐15 region.^2^ WDLPS and DDLPS are genetically characterized by MDM2 amplification. DDLPS is defined as the transition of WDLPS towards non‐lipogenic sarcoma.^3^ High‐grade pleomorphic sarcoma with a WDLPS component is a typical morphology of DDLPS.^2^ Liposarcomas are rare among children, representing about 2% of all pediatric soft tissue sarcomas.^4^ WDLPS and DDLPS subtypes, associated with MDM2 amplification, comprise approximately 13% of all pediatric liposarcomas.^5^

Some peripheral (extremities, trunk wall, head/neck) undifferentiated pleomorphic sarcomas without WDLPS components present MDM2 amplification detected using fluorescence in situ hybridization (FISH) analysis. These were similar to DDLPS in terms of their clinical courses and showed better prognosis than that of MDM2‐negative undifferentiated pleomorphic sarcomas.^6^ Recently, pleomorphic sarcomas with MDM2 amplification are considered to be DDLPS irrespective of the WDLPS components.^6^, ^7^, ^8^ In the study by a French group, patients with peripheral undifferentiated pleomorphic sarcomas harboring MDM2 amplification without WDLPS components were aged >45 years.^6^

Sarcomas with MDM2 amplification are rare among pediatric patients. We report a pediatric case of pleomorphic sarcoma of the head showing MDM2 amplification without a WDLPS component.

## 2 CASE PRESENTATION

**FIGURE 1:**
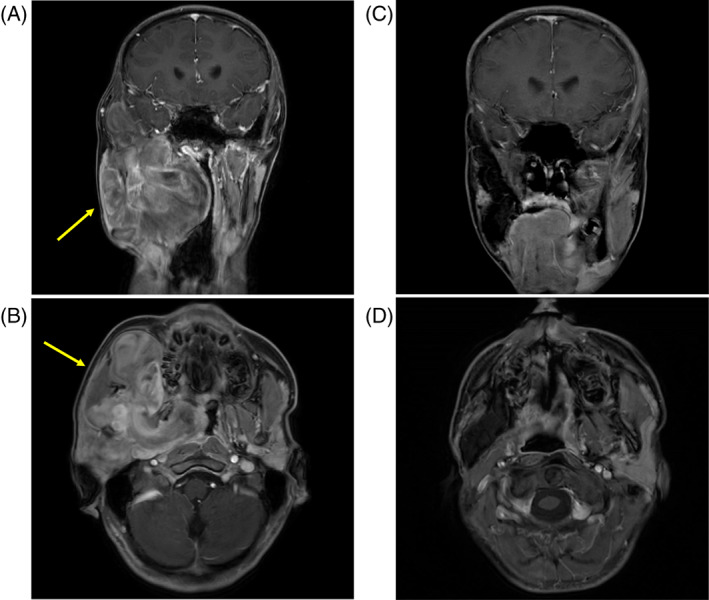
Contrast‐enhanced magnetic resonance imaging (MRI): (A and B) At diagnosis, the lobulated mass occupies the right side of the masticator space. The inside of the mass shows a non‐uniform contrast effect (Yellow arrow). (C and D) 3 years after completion of the therapy, no sign of recurrence is found.

A 14‐year‐old boy with a painless mandibular mass on the right side was referred to our hospital in March 2019. The mass developed approximately 4 months prior to referral and gradually enlarged in size. Contrast‐enhanced computed tomography (CT) revealed a lobulated mass (largest diameter, 12 cm) on the right side of the masticator space. Contrast‐enhanced magnetic resonance imaging (MRI) revealed iso signal intensity on T1‐weighted images and high signal intensity on T2‐weighted images in most areas of the mass. A non‐uniform contrast effect was noted inside the mass (Figure 1A,B), and fat signals were partially detected inside the mass. ^18^F‐fluorodeoxyglucose positron emission tomography with CT (^18^F‐FDG PET/CT) demonstrated a lobulated mass on the right side of the masticator space with minimal FDG activity (maximum standardized uptake value: SUVmax = 2.14) (Figure S1A). No other lesions were detected on contrast‐enhanced CT and ^18^F‐FDG PET/CT. Subsequently, we performed biopsy of the right mandibular mass.

**FIGURE 2:**
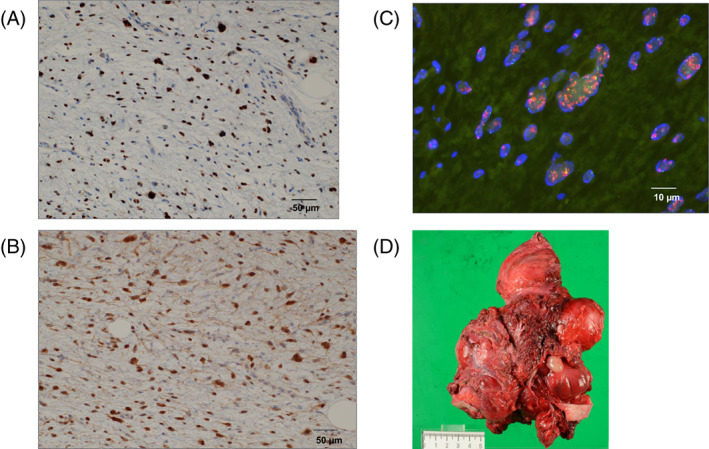
(A) Immunohistochemically, the atypical spindle‐shaped cells are positive for MDM2 on biopsy specimen (magnification, ×200). (B) Immunohistochemically, the atypical spindle‐shaped cells are positive for CDK4 on biopsy specimen (magnification, ×200). (C) MDM2 gene amplification is detected by FISH (red signals) on biopsy specimen (magnification, ×1000). The red signals, which are found in pairs per normal cell, are numerous due to MDM2 gene amplification. (D) Gross appearance of a lobulated mass, measuring 14.5 × 13.5 × 7 cm.

Histologically, hematoxylin–eosin staining showed proliferation of atypical spindle‐shaped cells with fibro‐collagenous stroma. Pleomorphic tumor cells and adipocytes with minimal atypia were also observed. Immunohistochemically, atypical spindle‐shaped cells stained positive for MDM2 and cyclin‐dependent kinase 4 (CDK4) (Figure 2A,B), and negative for epithelial membrane antigen, mucin 4, alpha‐smooth muscle actin, S100 protein, and myogenin. MDM2 gene amplification was detected using FISH (Figure 2C). The tumor was histologically categorized as Grade 2 according to the Fédération Nationale des Centres de Lutte Contre le Cancer (FNCLCC) system, and the stage was classified as Stage IIIB based on the American Joint Committee on Cancer staging system. Despite the absence of a WDLPS component, the patient was diagnosed with DDLPS owing to MDM2 gene amplification. Because the biopsy specimen was a small portion of the entire tumor, a WDLPS component might have been present in the remaining area.

Complete surgical resection was the best treatment; however, it was difficult because the tumor occupied the masticator space. Additionally, we considered that surgical margins might be positive even if macroscopic complete surgical resection was possible. Therefore, we decided to administer postoperative radiotherapy if there was microscopic residual disease. Although the effect of chemotherapy might be limited, we administered chemotherapy to shrink the tumor as much as possible before surgery. Chemotherapy was administered using doxorubicin (25 mg/m^2^/day on days 1 and 2) and ifosfamide (2 g/m^2^/day on days 1–5). The tumor did not shrink after three courses of chemotherapy. Moreover, no change was observed in the FDG activity (SUVmax = 1.92) as per ^18^F‐FDG PET/CT (Figure S1B). Although chemotherapy was not effective, we performed complete surgical resection of the tumor.

Macroscopic examination revealed a lobulated whitish‐yellow mass measuring 14.5 × 13.5 × 7 cm (Figure 2D). On microscopic examination, the completely resected specimen showed no evidence of a WDLPS component, and most tumor cells were viable. Although the lobulated mass was covered with a thin fibrous capsule, tumor cells infiltrated the surrounding fatty tissue outside the capsule in some areas (Figure S2). Following the 2013 World Health Organization (WHO) classification of tumors, the tumor was not DDLPS due to the absence of a WDLPS component. However, as per the suggestion of a French group,^6^ we diagnosed the patient with DDLPS owing to the MDM2 gene amplification for treatment.

Although macroscopic examination showed complete resection of the tumor, the surgical margin was positive. Therefore, postoperative proton‐beam radiotherapy (50.4 Gy; 1.8 Gy/day) was performed against the microscopic residual disease. 3 years following therapy completion, the patient did not show any sign of recurrence (Figure 1C,D). Currently, the patient chews on the left side and has good oral intake. The patient is a member of his high school softball team and has a Karnofsky performance status score of 100.

## 3 DISCUSSION

Our patient was clinically diagnosed with DDLPS owing to the positive immunostaining results for MDM2 and CDK4 and MDM2 gene amplification. The overall MDM2 amplification frequency in all human cancers is low, varying between 3.5% and 4.4%.^3^ However, its amplification frequency in soft tissue tumors is very high, particularly in WDLPS and DDLPS.^9^ CDK4, which is located at 12q13‐14, is a key regulator of the G1/S cell cycle checkpoint, and it increases cell proliferation.^1^, ^2^ DDLPS is associated with high‐amplification of MDM2 and CDK4, which often leads to overexpression of MDM2 and CDK4; therefore, immunohistochemistry staining for MDM2 and CDK4 can also be used for the diagnosis of DDLPS.^10^, ^11^ Immunohistochemistry analysis has a sensitivity of 95% and 92% and a specificity of 81% and 95% for MDM2 and CDK4, respectively in the diagnosis of DDLPS.^1^ Detection of MDM2 amplification by FISH can be used to confirm the diagnosis of DDLPS, especially in limited biopsy samples.^7^

Variable FDG uptake in ^18^F‐FDG PET/CT is reported in DDLPS.^12^, ^13^, ^14^ The mean SUVmax in previous reports was 16.3 ± 11.4 (range 3.4–39.6)^12^ and 9.23 ± 7.63 (range 2.3–29.5).^13^ Previous studies reported an association between higher FNCLCC grade and higher SUVmax in retroperitoneal DDLPS.^14^, ^15^, ^16^ Further, they showed that SUV max >5.3 or 4.8 can be used to predict FNCLCC Grade 3. Increased SUVmax was also associated with poor overall survival.^15^, ^16^ In our patient, the SUVmax was 2.14 at diagnosis, with FNLCC Grade 2 tumor following examination of the biopsy specimen. This was consistent with previous findings regarding the association between SUVmax and pathological grade.

Complete surgical resection remains the primary and most prevalent treatment for DDLPS.^17^, ^18^ Primary radiotherapy is commonly used preoperatively to shrink retroperitoneal and intra‐abdominal DDLPS,^17^ and adjuvant radiotherapy is effective in controlling microscopic residual disease after surgical resection.^4^ Radiotherapy (preoperative or postoperative) is associated with better overall survival in patients with retroperitoneal sarcoma than surgery alone.^19^ Further, preoperative radiotherapy is associated with a greater risk of wound complications than postoperative radiotherapy in soft tissue sarcoma of the limbs.^20^ Some reports indicated minimum benefit of chemotherapy for DDLPS.^21^ Other reports revealed that chemotherapy for DDLPS was effective in shrinking tumors.^22^ Dose‐intensive ifosfamide plus doxorubicin chemotherapy is commonly used for DDLPS.^18^, ^22^ This regimen is also used to treat pediatric patients with unresected intermediate‐risk and high‐risk soft‐tissue sarcomas.^23^ The partial response rate of this regimen is approximately 30% in patients with DDLPS.^22^ In a case report, a patient with retroperitoneal DDLPS underwent six courses of this regimen preoperatively, and the tumor size was considerably reduced.^24^ In this report, the pathological findings indicated a 99% disappearance of tumor cells in the resected specimen.^24^

We considered complete surgical resection as the best treatment for our patient. However, based on the location, size, and extent of the tumor, we considered that surgical margins might be positive even if macroscopic complete surgical resection was possible. Therefore, we decided to administer postoperative radiotherapy if there was microscopic residual disease. Although the partial response rate of chemotherapy with doxorubicin and ifosfamide is approximately 30%,^22^ this regimen may induce the shrinkage of the tumor a little. Hence, we chose chemotherapy with doxorubicin and ifosfamide to shrink the tumor as much as possible before surgery. However, it was ineffective for our patient, and we subsequently performed the surgery. The surgical margin was positive on microscopic examination. Generally, 60–70 Gy of external beam radiotherapy is administered for microscopic surgical margin‐positive high‐grade soft tissue sarcoma of the extremity.^25^ Regarding postoperative radiotherapy for DDLPS, 60 Gy is used in patients with a positive surgical margin.^26^, ^27^ Considering the tolerable dose of the optic nerve, 50.4 Gy was used in postoperative proton‐beam radiotherapy against microscopic residual disease in our patient.

We report a rare pediatric case of pleomorphic sarcoma of the head showing MDM2 amplification by FISH. Following the World Health Organization (WHO) classification of tumors, the tumor of our patient was not DDLPS due to the absence of a WDLPS component. Molecular profiling could help diagnose in unusual pediatric cases; hence, it should be considered to assist with diagnosing rare pediatric sarcomas in the near future.^5^, ^8^, ^9^ Based on our study outcome, a combination of macroscopic surgical resection with adjuvant postoperative radiotherapy could establish a good prognosis in pediatric patients with MDM2‐ amplified pleomorphic sarcoma refractory to neoadjuvant chemotherapy. This study has a limitation because it comprised just one case report. Owing to the rarity of such sarcomas in pediatric patients, there is insufficient knowledge regarding the ideal management and treatment strategies for these tumors. Further studies are needed to identify the optimal therapy.

## AUTHOR CONTRIBUTIONS

**Mitsuko Akaihata:** Conceptualization (equal); writing – original draft (equal). **Ikuko Takahashi:** Conceptualization (supporting). **Yuko Kakuda:** Conceptualization (supporting); visualization (equal). **Takuya Kawata:** Conceptualization (supporting); visualization (equal). **Takashi Mukaigawa:** Conceptualization (supporting). **Testuro Onitsuka:** Conceptualization (supporting). **Shigeyuki Murayama:** Conceptualization (supporting). **Yuji Ishida:** Conceptualization (lead); supervision (equal); writing – review and editing (equal).

## CONFLICT OF INTEREST

The authors have stated explicitly that there are no conflicts of interest in connection with this article.

## ETHICS STATEMENT

Informed consent was obtained from the patient for the publication of this case report.

## Supporting information


**Figure S1.** ^18^F‐fluorodeoxyglucose positron emission tomography with computed tomography (^18^F‐FDG PET/CT): (A)Image at diagnosis. A mass at the right side of the masticator space shows minimal FDG activity (SUVmax = 2.14). Two punctate accumulations (SUVmax = 3.61), which are consistent with lymph nodes, are found on the underside of the mass. These accumulations are considered as reactive lymph node enlargement. (B) Pre‐surgery image. FDG activity at the right side of the masticator space is similar to that at diagnosis (SUVmax = 1.92).

**Figure S2.** Hematoxylin–eosin staining shows that pleomorphic tumor cells (Yellow arrow) infiltrate outside the capsule (Blue arrows) on the surgically resected specimen (magnification, ×40). The surgical margin is positive.

## Data Availability

Data sharing is not applicable to this article as no new data were created or analyzed in this study.

## References

[1] Coindre JM , Pédeutour F , Aurias A . Well‐differentiated and dedifferentiated liposarcomas. Virchows Arch. 2010;456:167‐179. 19688222 10.1007/s00428-009-0815-x

[2] Lee ATJ , Thway K , Huang PH , Jones RL . Clinical and molecular spectrum of liposarcoma. J Clin Oncol. 2018;36:151‐159. 29220294 10.1200/JCO.2017.74.9598 PMC5759315

[3] Sciot R . MDM2 amplified sarcomas: a literature review. Diagnostics (Basel). 2021;11:11. 10.3390/diagnostics11030496 PMC8001728 33799733

[4] Huh WW , Yuen C , Munsell M , et al. Liposarcoma in children and young adults: a multi‐institutional experience. Pediatr Blood Cancer. 2011;57:1142‐1146. 21394894 10.1002/pbc.23095 PMC3134599

[5] Ameloot E , Cordier F , Van Dorpe J , Creytens D . Update of pediatric Lipomatous lesions: a clinicopathological, immunohistochemical and molecular overview. J Clin Med. 2022;11:11. 10.3390/jcm11071938 PMC8999862 35407546

[6] Le Guellec S , Chibon F , Ouali M , et al. Are peripheral purely undifferentiated pleomorphic sarcomas with MDM2 amplification dedifferentiated liposarcomas? Am J Surg Pathol. 2014;38:293‐304. 24525499 10.1097/PAS.0000000000000131

[7] Hornick JL . Subclassification of pleomorphic sarcomas: how and why should we care? Ann Diagn Pathol. 2018;37:118‐124. 30340082 10.1016/j.anndiagpath.2018.10.006

[8] Oda Y , Yamamoto H , Kohashi K , et al. Soft tissue sarcomas: from a morphological to a molecular biological approach. Pathol Int. 2017;67:435‐446. 28759137 10.1111/pin.12565

[9] Lu J , Wood D , Ingley E , Koks S , Wong D . Update on genomic and molecular landscapes of well‐differentiated liposarcoma and dedifferentiated liposarcoma. Mol Biol Rep. 2021;48:3637‐3647. 33893924 10.1007/s11033-021-06362-5

[10] Binh MB , Sastre‐Garau X , Guillou L , et al. MDM2 and CDK4 immunostainings are useful adjuncts in diagnosing well‐differentiated and dedifferentiated liposarcoma subtypes: a comparative analysis of 559 soft tissue neoplasms with genetic data. Am J Surg Pathol. 2005;29:1340‐1347. 16160477 10.1097/01.pas.0000170343.09562.39

[11] Tan MC , Brennan MF , Kuk D , et al. Histology‐based classification predicts pattern of recurrence and improves risk stratification in primary retroperitoneal sarcoma. Ann Surg. 2016;263:593‐600. 25915910 10.1097/SLA.0000000000001149 PMC4619189

[12] Baffour FI , Wenger DE , Broski SM . (18)F‐FDG PET/CT imaging features of lipomatous tumors. Am J Nucl Med Mol Imaging. 2020;10:74‐82. 32211221 PMC7076300

[13] Parkes A , Urquiola E , Bhosale P , et al. PET/CT imaging as a diagnostic tool in distinguishing well‐differentiated versus dedifferentiated liposarcoma. Sarcoma. 2020;2020:8363986. 32565716 10.1155/2020/8363986 PMC7285404

[14] Li CP , Liu DN , Zhou NN , et al. Prediction of histologic subtype and FNCLCC grade by SUVmax measured on (18)F‐FDG PET/CT in patients with retroperitoneal liposarcoma. Contrast Media Mol Imaging. 2021;2021:7191363. 33505228 10.1155/2021/7191363 PMC7806371

[15] Subramaniam S , Callahan J , Bressel M , et al. The role of (18) F‐FDG PET/CT in retroperitoneal sarcomas‐a multicenter retrospective study. J Surg Oncol. 2021;123:1081‐1087. 33444466 10.1002/jso.26379

[16] Jo SJ , Kim KD , Lim SH , et al. The role of preoperative (18)F‐fluorodeoxyglucose positron emission tomography/computed tomography in retroperitoneal sarcoma. Front Oncol. 2022;12:868823. 35712466 10.3389/fonc.2022.868823 PMC9197420

[17] Gootee J , Aurit S , Curtin C , Silberstein P . Primary anatomical site, adjuvant therapy, and other prognostic variables for dedifferentiated liposarcoma. J Cancer Res Clin Oncol. 2019;145:181‐192. 30361927 10.1007/s00432-018-2777-3 PMC11810416

[18] Gahvari Z , Parkes A . Dedifferentiated liposarcoma: systemic therapy options. Curr Treat Options Oncol. 2020;21:15. 32026050 10.1007/s11864-020-0705-7

[19] Nussbaum DP , Rushing CN , Lane WO , et al. Preoperative or postoperative radiotherapy versus surgery alone for retroperitoneal sarcoma: a case‐control, propensity score‐matched analysis of a nationwide clinical oncology database. Lancet Oncol. 2016;17:966‐975. 27210906 10.1016/S1470-2045(16)30050-X

[20] O'Sullivan B , Davis AM , Turcotte R , et al. Preoperative versus postoperative radiotherapy in soft‐tissue sarcoma of the limbs: a randomised trial. Lancet. 2002;359:2235‐2241. 12103287 10.1016/S0140-6736(02)09292-9

[21] Jones RL , Fisher C , Al‐Muderis O , Judson IR . Differential sensitivity of liposarcoma subtypes to chemotherapy. Eur J Cancer. 2005;41:2853‐2860. 16289617 10.1016/j.ejca.2005.07.023

[22] Livingston JA , Bugano D , Barbo A , et al. Role of chemotherapy in dedifferentiated liposarcoma of the retroperitoneum: defining the benefit and challenges of the standard. Sci Rep. 2017;7:11836. 28928422 10.1038/s41598-017-12132-w PMC5605500

[23] Spunt SL , Million L , Chi YY , et al. A risk‐based treatment strategy for non‐rhabdomyosarcoma soft‐tissue sarcomas in patients younger than 30 years (ARST0332): a Children's Oncology Group prospective study. Lancet Oncol. 2020;21:145‐161. 31786124 10.1016/S1470-2045(19)30672-2 PMC6946838

[24] Yokoyama Y , Nishida Y , Ikuta K , Nagino M . A case of retroperitoneal dedifferentiated liposarcoma successfully treated by neoadjuvant chemotherapy and subsequent surgery. Surg Case Rep. 2020;6:105. 32448975 10.1186/s40792-020-00865-2 PMC7246274

[25] Alektiar KM , Velasco J , Zelefsky MJ , Woodruff JM , Lewis JJ , Brennan MF . Adjuvant radiotherapy for margin‐positive high‐grade soft tissue sarcoma of the extremity. Int J Radiat Oncol Biol Phys. 2000;48:1051‐1058. 11072162 10.1016/s0360-3016(00)00753-7

[26] Nimura F , Nakasone T , Matsumoto H , et al. Dedifferentiated liposarcoma of the oral floor: a case study and literature review of 50 cases of head and neck neoplasm. Oncol Lett. 2018;15:7681‐7688. 29740489 10.3892/ol.2018.8274 PMC5934721

[27] Kito M , Yoshimura Y , Isobe K , et al. Clinical outcome of dedifferentiated liposarcoma in the extremities: a retrospective case series of 7 patients. J Orthop Sci. 2016;21:673‐677. 27317086 10.1016/j.jos.2016.05.006

