# Supplementary figures and images for: Head pleomorphic sarcoma showing murine double minute 2 amplification without a well‐differentiated liposarcoma component in a pediatric patient
Source: Cancer Rep (Hoboken). 2022 Dec 26;6(2):e1774. doi: 10.1002/cnr2.1774 (PMC9939988; doi:10.1002/cnr2.1774)

A

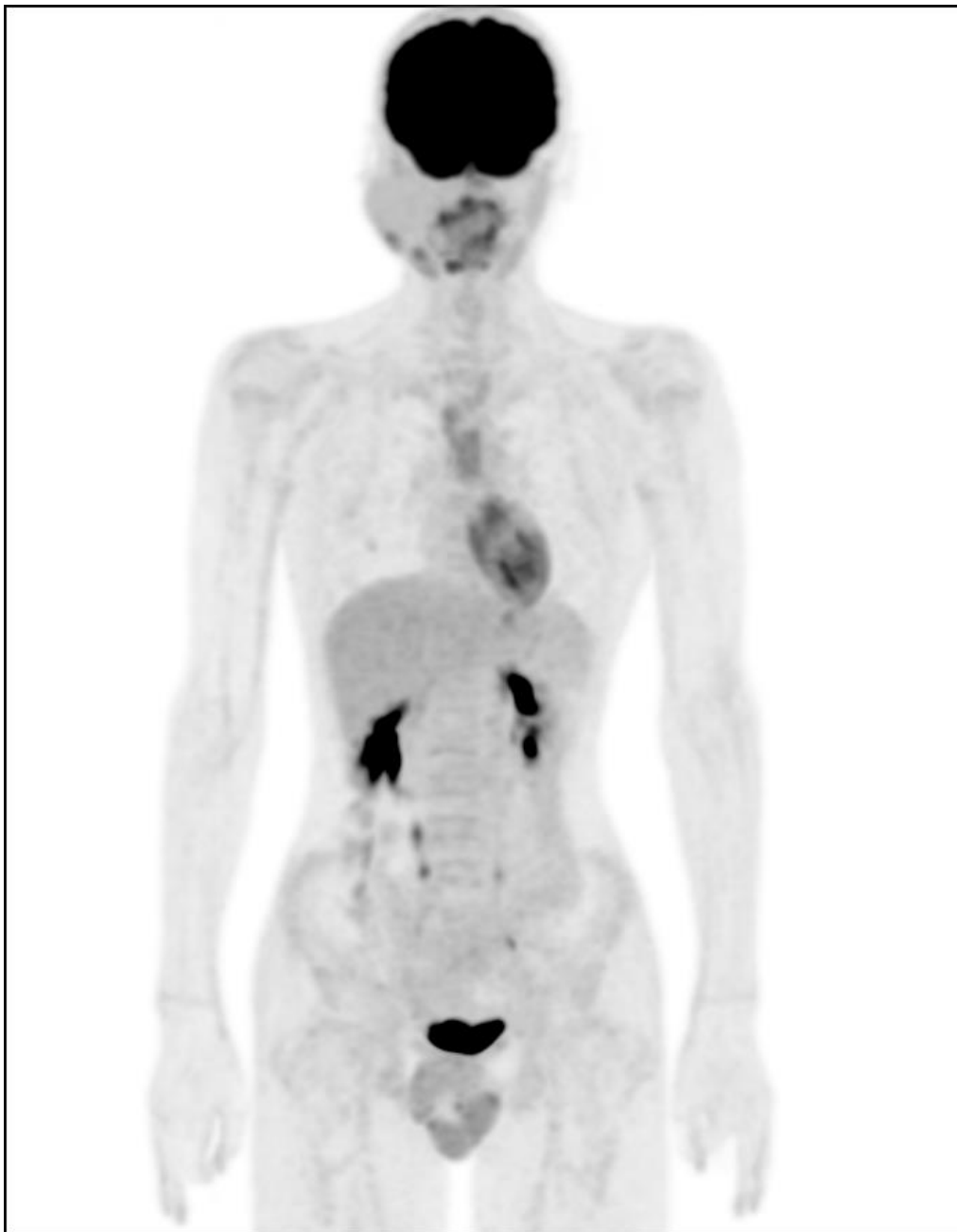

B

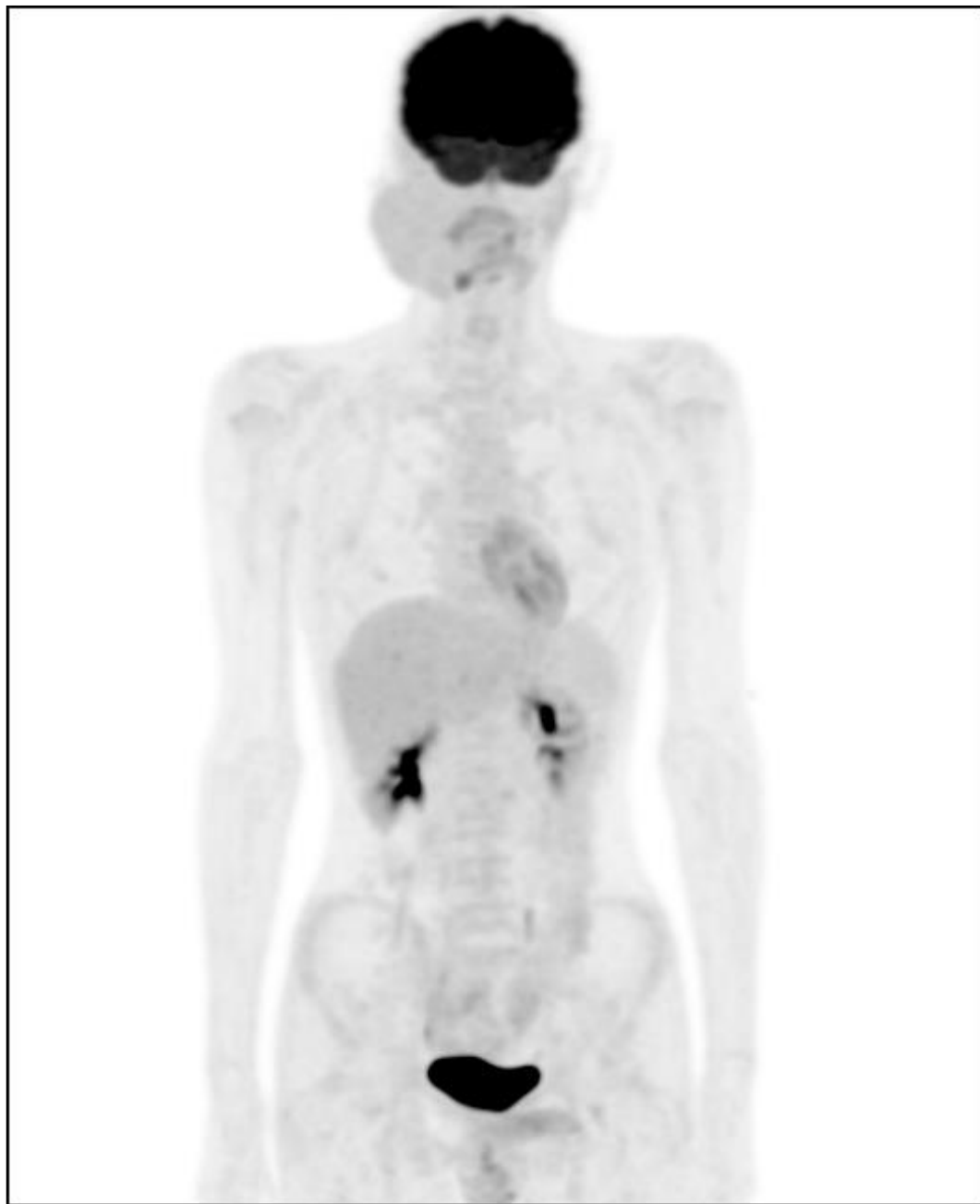

Supplement: Supplementary file 1 — Figure S1. 18F‐fluorodeoxyglucose positron emission tomography with computed tomography (18F‐FDG PET/CT): (A)Image at diagnosis. A mass at the right side of the masticator space shows minimal FDG activity (SUVmax = 2.14). Two punctate accumulations (SUVmax = 3.61), which are consistent with lymph nodes, are found on the underside of the mass. These accumulations are considered as reactive lymph node enlargement. (B) Pre‐surgery image. FDG activity at the right side of the masticator space is similar to that at diagnosis (SUVmax = 1.92). [file CNR2-6-e1774-s001.pdf]

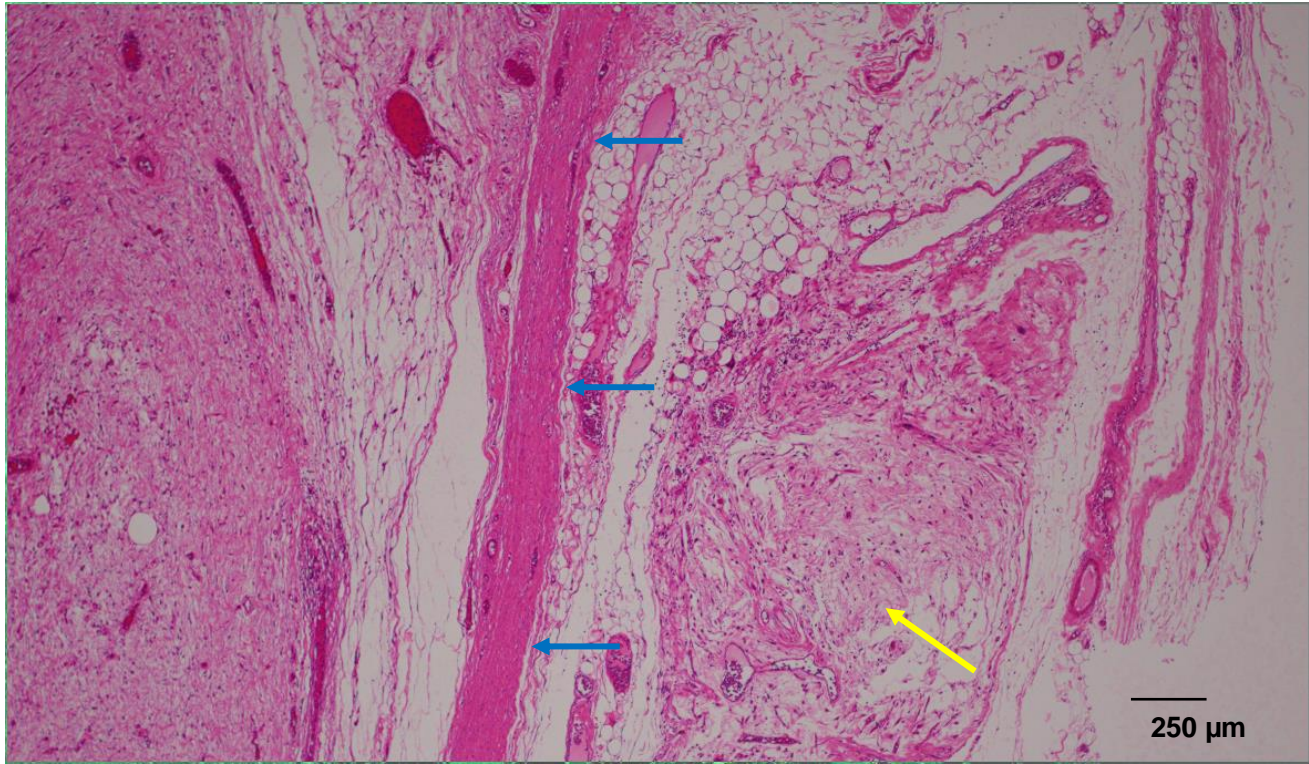

Supplement: Supplementary file 2 — Figure S2. Hematoxylin–eosin staining shows that pleomorphic tumor cells (Yellow arrow) infiltrate outside the capsule (Blue arrows) on the surgically resected specimen (magnification, ×40). The surgical margin is positive. [file CNR2-6-e1774-s002.pdf]
